# Promotion on Acetone Sensing of Single SnO_2_ Nanobelt by Eu Doping

**DOI:** 10.1186/s11671-017-2177-7

**Published:** 2017-06-12

**Authors:** Weiwu Chen, Zhaojun Qin, Yingkai Liu, Yan Zhang, Yanbo Li, Si Shen, Zhiming M. Wang, Hai-Zhi Song

**Affiliations:** 1 Institute of Fundamental and Frontier Sciences, University of Electronic Science and Technology of China, Chengdu, 610054 People’s Republic of China; 2 Institute of Physics and Electronic Information Technology, Yunnan Normal University, Kunming, 650500 People’s Republic of China; 3 Beijing Institute of Nanoenergy and Nanosystems, Chinese Academy of Sciences, Beijing, 100083 People’s Republic of China; 4 Southwest Institute of Technical Physics, Chengdu, 610041 People’s Republic of China

**Keywords:** Eu-doped SnO_2_, Single nanobelt, Acetone sensor

## Abstract

SnO_2_ nanobelts (NBs) have unique structural and functional properties which attract great attention in gas detecting. In this work, Eu doping is adopted to improve the gas sensitivity of pure SnO_2_, especially to enhance the response to one single gas. The Eu-doped SnO_2_ NBs, pure-SnO_2_ NBs, and their single NB devices are fabricated by simple techniques. The sensing properties of the two sensors have been experimentally investigated. It is found that the two sensors possess long-term stability with rapid response performance, and Eu doping improves the electronic performance and the gas-sensing response, particularly to acetone. In addition, the effects aroused by Eu have been theoretically calculated, which indicates that Eu doping enhances the sensing performance of SnO_2_. Consequently, Eu-doped SnO_2_ NBs show great potential applications in the detection of acetone.

## Background

With the development of industry, as an important aspect of environmental problems, the leakage of harmful gases becomes more and more eye-catching. Many efforts of improving the gas sensor performance have been made in order to detect and monitor those gases. Excellent accomplishments have been reached in the field of gas sensor due to the remarkable advancement in novel nanomaterials [1–3].

Among various shapes of nanomaterials, nanobelt is a promising choice in gas sensing application [4, 5] since it could bear a large specific surface area, crystallographic perfection, and great electron transport properties. For instance, Khiabani et al. have reported that In_2_O_3_ NBs have excellent gas sensitive properties for NO_2_ [6]. As to metal oxide semiconductors, their susceptibility coupled with stabilization makes it very applicable to the detection of various gases [7–9]. As an n-type wide-bandgap semiconductor, SnO_2_ with a high gas-sensitive response to a variety of gases has attracted worldwide attention [10–12]. It has been proved by Huang et al. that SnO_2_ nanorod arrays take the possession of unique performance as a hydrogen sensor [13]. In such materials, rare metal doping is often used to improve the sensitivity, especially to one single gas [14, 15]. As a typical rare earth metal, it has been proved to be effective for Eu to improve the sensing performance of various materials [16–19]. Especially, Hao et al. have testified the positive effects of Eu doping on the sensing and electrical conductivity of Eu-based metal-organic framework [20]. However, to the best of our knowledge, there are still very few studies about Eu doping effects on the gas-sensitive properties so far. Thus, it is a requisite to explore the gas-sensing properties of Eu-doped-SnO_2_ nanobelts (Eu-SnO_2_ NBs) to make progress in the sensitivity of pure-SnO_2_ nanobelts (SnO_2_ NBs).

In this work, we have made the synthesis of SnO_2_ NBs and Eu-SnO_2_ NBs by thermal evaporation method with simple conditions, low cost, and accessibility. The sensitivity of SnO_2_ NBs and Eu-SnO_2_ NBs to four gases was measured, and it is demonstrated that the Eu-SnO_2_ NB sensor owns a higher response, especially to acetone. The conceivable mechanism was proposed on the basis of theoretical calculations. It turns out that Eu-SnO_2_ NBs reveal great potential in acetone-sensing applications.

## Methods

The synthesis of NBs was conducted in a horizontal tube furnace (HTF) with an alundum tube. The raw materials which provided Sn element were pure SnO_2_ powders, and Eu ions were supplied by pure Eu(O_2_CCH_3_)_3_ powders with a mass ratio of 19:1 for the preparation of the doped NBs. Then, the ingredients were filled into a ceramic boat being laid in the middle of the HTF and a silicon wafer plated with 10 nm Au film was positioned downstream 20 cm far away from the vessel. Subsequently, HTF was rinsed by argon, and then the temperature of the central region climbed up to 1355 °C with a ramp-up of 10 °C/min and then was kept at 1355 °C for 120 min. The flow of argon as carrier gas was at 20 sccm in the meantime, and the internal pressure was maintained at 200 torr by means of a mechanical pump. At last, the temperature declined naturally and the required NBs were obtained.

The specimens were characterized by X-ray diffraction (XRD) (D/max-3B Rigaku with Cu-Kα radiation, *λ* = 0.15406 nm), scanning electron microscopy (SEM) (Quanta 200 FEG, FEI Company), energy dispersive X-ray spectroscopy (EDS) (Octane Super, EDAX), X-ray photoelectron spectroscopy (XPS) (PHI 5000 Versaprobe, UlVAC-PHI), and high-resolution transmission electron microscopy (HRTEM) attached with the selected area electron diffraction (SAED) (Tecnai G_2_ Transmission Electron Microscope, 200 kV).

**Fig. 1:**
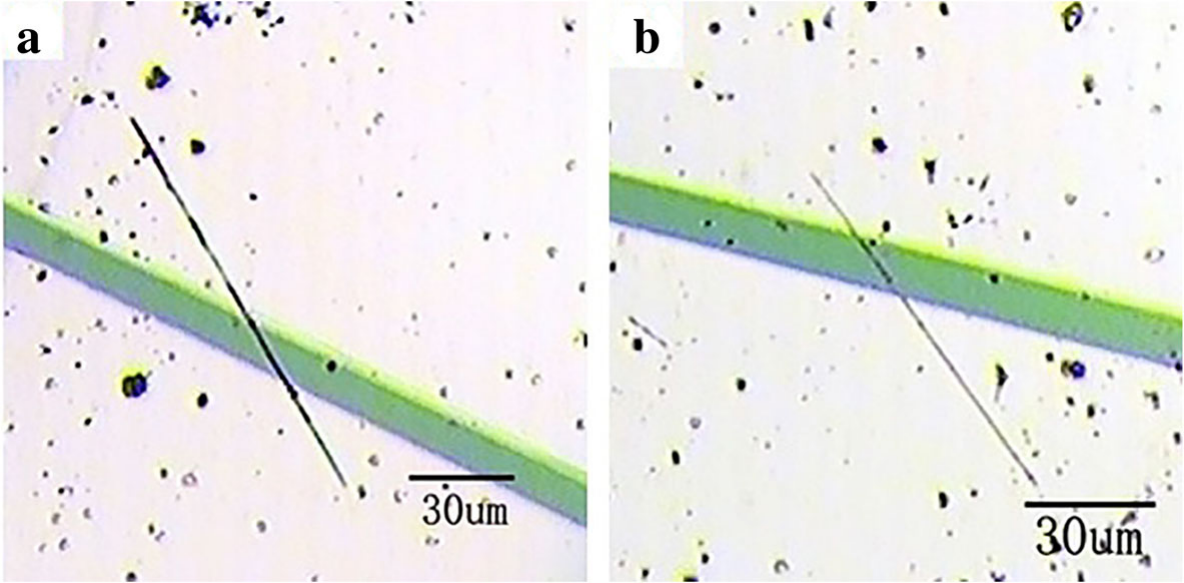
Optical microscope photos of **a** Eu-SnO_2_ NB and **b** SnO_2_ NB devices

The single nanobelt devices were manufactured by dual-ion beam deposition (LDJ-2a-F100-100 series) with an aid of the mesh-grid mask. First of all, a few nanobelts were dissolved into ethanol liquid to prepare a floating liquid, and then the floating liquid was dripped down to the surface of silicon wafer uniformly, which could result in the uniform distribution of nanobelts on the surface of silicon wafer. Thereafter, Ti (8 nm) and Au (80 nm) electrodes were deposited on the substrate in the conditions of the pressure of 2.2 × 10^−2^ Pa and argon ion flow of 10 mA/cm^2^. After these, the preparation has been accomplished and the measurements would be conducted by Keithley 4200 SCS. Figure 1 shows optical microscope photos of two single nanobelt devices, manifesting that the lengths/widths of the doped and pure nanobelts are about 118.13/1.47 and 83.48/0.87 μm, respectively.

**Fig. 2:**
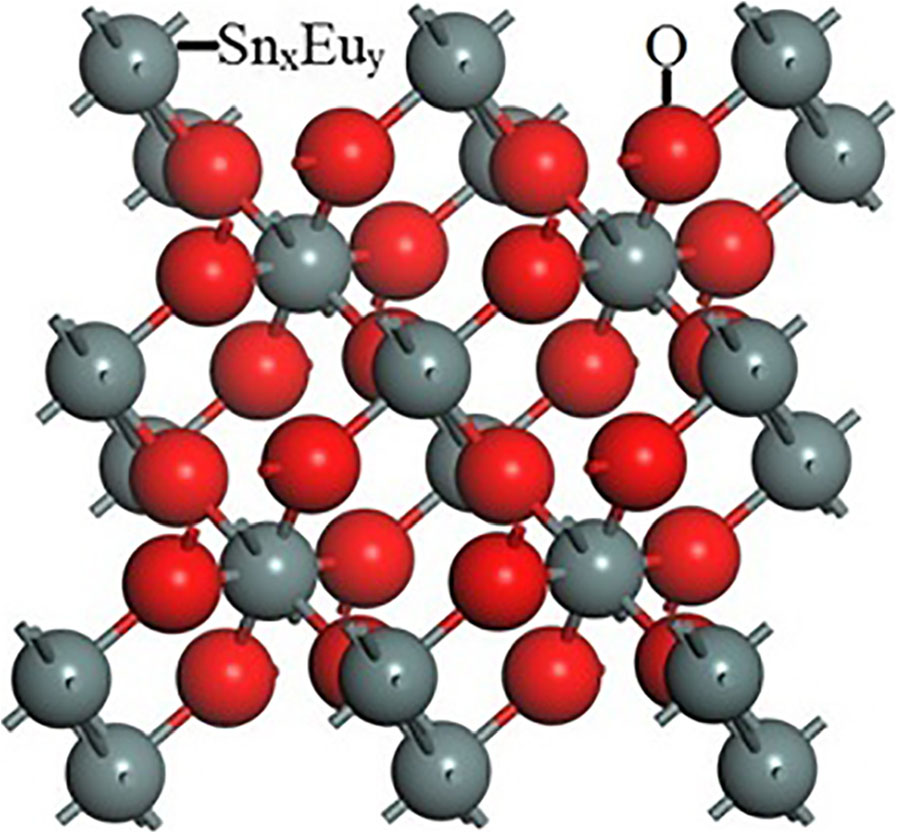
Structure diagram of Sn_x_Eu_y_O_16_ (*x* = 8, *y* = 0 for SnO_2_ and *x* = 7.94, *y* = 0.06 for Eu-SnO_2_)

The calculations about the band structure and density of states of these two nanobelts were made by CASTEP module of Materials Studio. According to the density functional theory (DFT), PBE function of generalized gradient approximation (GGA) was used to amend the exchange-related potential and optimize the crystal structure [21]. SnO_2_ belongs to a body-centered tetragonal structure, whose symmetry is D_4h−14_ [22]. Then, we built a 2 × 2 × 1 supercell structure and substituted Sn atoms into the mixture of 93.75% Sn and 6.25% Eu to get the uniform dopant effect corresponding to Sn_7.94_Eu_0.06_O_16_, as shown in Fig. 2. The energy cutoff, k-point set, and self-consistent field tolerance were set to be 340 eV, 3 × 3 × 8, and 1.0 × 10^−6^ eV, respectively.

## Results and discussion

**Fig. 3:**
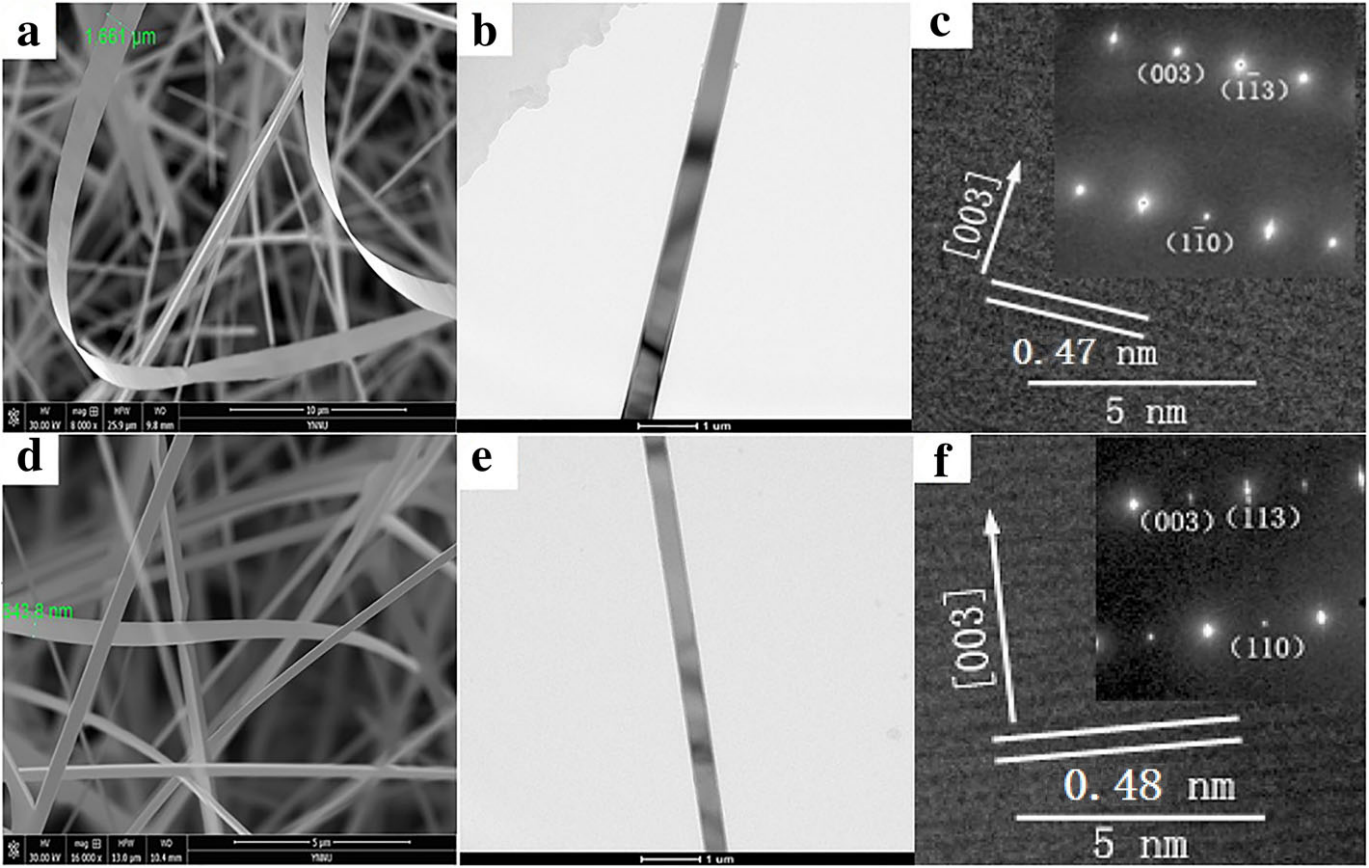
The morphology images of Eu-SnO_2_ NB and SnO_2_ NB. **a** SEM, **b** TEM, and **c** HRTEM images of Eu-SnO_2_ NB; **d** SEM, **e** TEM, and **f** HRTEM images of SnO_2_ NB

The SEM images in Fig. 3a, d show that the widths of Eu-doped and pure SnO_2_ NBs with regular morphology are 1.661 μm and 543.8 nm, respectively. The TEM images in Fig. 3b, e reveal that the Eu-doped and pure SnO_2_ nanobelts are homogeneous with no remarkable surface defects. Their corresponding HRTEM and SAED patterns in Fig. 3c, f indicate that their growths are both directed along [0 0 3], since the measured inter-planar spacing of 0.47 and 0.48 nm corresponds to the spacing of the (0 0 3) planes. These diffraction spots formed a rectangular array in conformity with tetragonal structure SnO_2_ which could manifest crystallographic perfection.

**Fig. 4:**
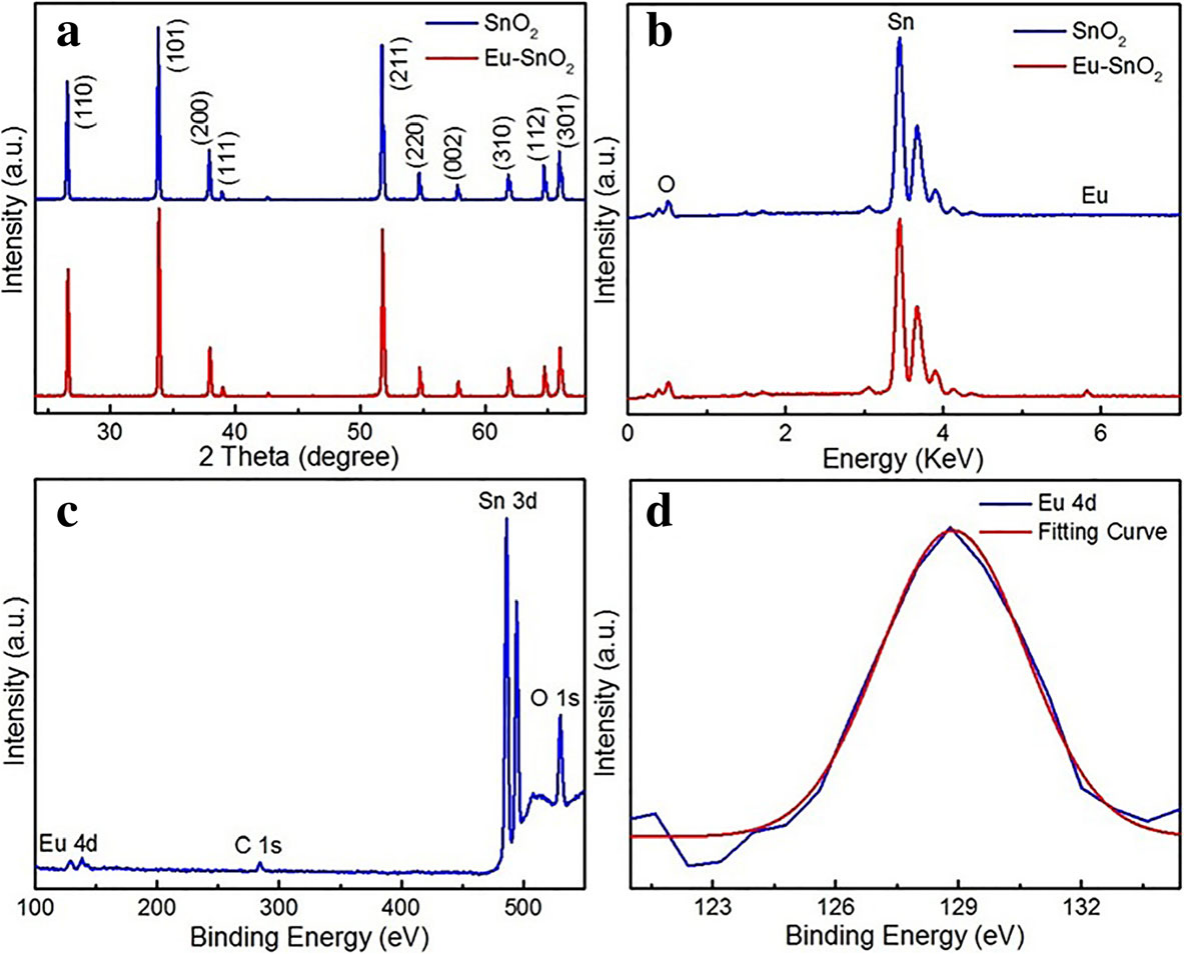
**a** XRD, **b** EDS, and **c** XPS spectra of Eu-SnO_2_ and pure NBs; **d** High-resolution XPS spectra for Eu 4d

The XRD spectra in Fig. 4a show that all diffraction peaks of Eu-SnO_2_ and SnO_2_ NBs can be indexed as the tetragonal rutile SnO_2_ phase (JCPDS card No.77-0450) with a = b = 0.473 nm and c = 0.318 nm. At the same time, it is revealed that the diffraction peaks of the admixtures move toward low angles, and it could be proved that Eu has been doped into the lattice. This is reasonable, considering that the radius of Eu ion (94.7 pm) is larger than that of Sn ion (69 pm). The EDS spectra in Fig. 4b can confirm it that Eu ions have been doped into SnO_2_ NBs. Based on the EDS data, it could be deduced that the ratios of Sn and O ions are 1:1.68 in Eu-SnO_2_ NBs and 1:1.76 in SnO_2_ NBs, indicating that there exist oxygen vacancies.

As shown in Fig. 4c, XPS spectrum displays that SnO_2_ NBs contain Sn 3d, O 1s, Eu 4d, and C 1s states. It is indicative of the successful doping of Eu into SnO_2_. In Fig. 4d, the Eu 4d peak having great symmetry could be well fitted by a Gaussian spectrum. It implies that there is only Eu 4d_5/2_ located in a 128.9 eV state arising from trivalent Eu, so the main Eu element in Eu-SnO_2_ NBs is Eu^3+^.

**Fig. 5:**
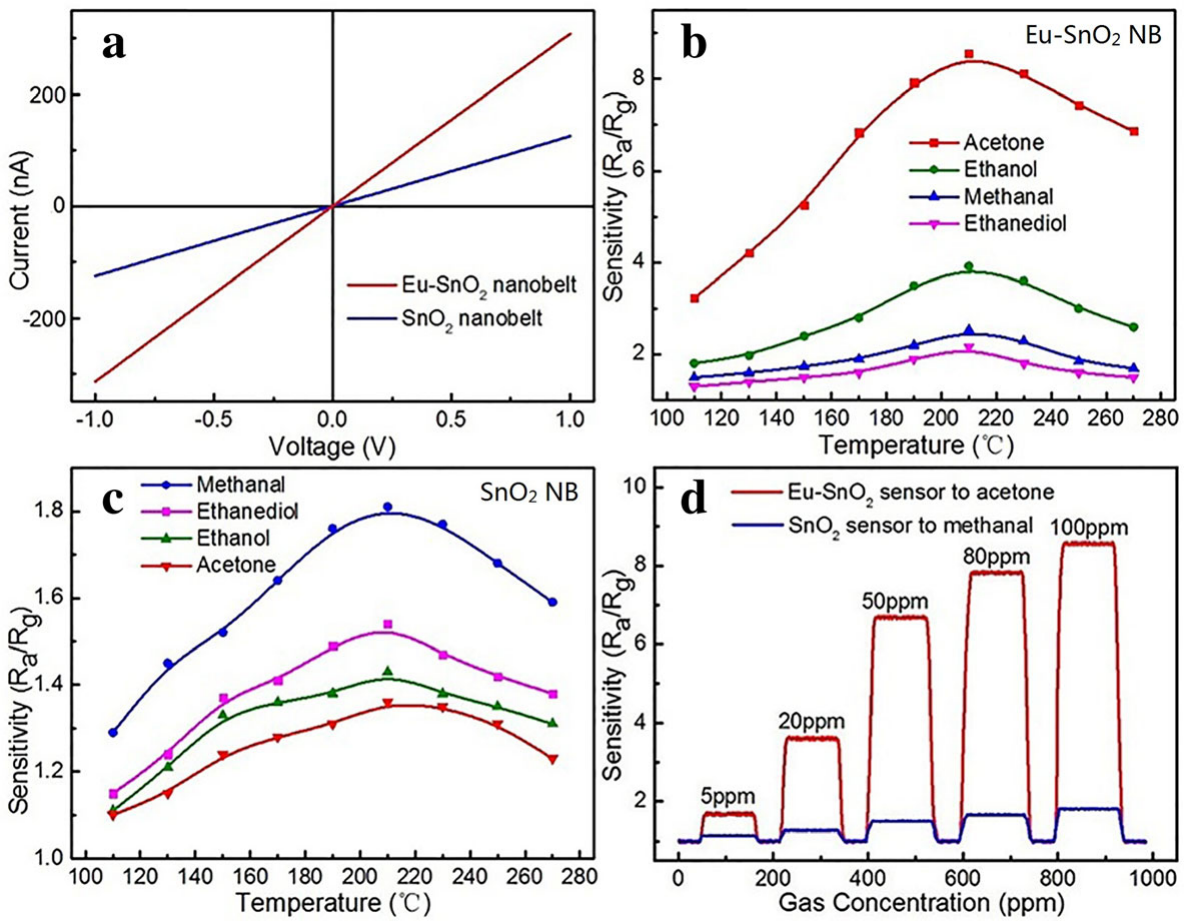
**a** I–V curves. **b** Response versus temperature curves of Eu-SnO_2_ NB. **c** Response versus temperature curves of SnO_2_ NB. **d** Chemical resistance response

From the I–V curves of the two sensors in Fig. 5a, it is known that the two sensors both have good ohmic contact but a noteworthy disparity in resistance. The resistance is found to be about 3.25 MΩ for Eu-SnO_2_ NBs and 7.97 MΩ for SnO_2_ NBs. Obviously, Eu doping has been successful in improving the conductivity of SnO_2_ NBs. The sensitivity is defined as R_a_/R_g_, where R_a_ is the resistance in air and R_g_ is the resistance in target gas. With a reducing gas circulating inside, the tendency of change of the resistance of Eu-SnO_2_ NB is the same as that of SnO_2_ NB, which indicates that Eu-SnO_2_ NB is a n-type semiconductor. As depicted in Fig. 5b, c, the gas responses of Eu-doped and pure sensors to 100 ppm of acetone, ethanol, methanal, and ethanediol at different temperatures have been investigated. The optimum working temperature of them is 210 °C. For different target gases, acetone, ethanol, methanol, and ethanediol, the highest sensitivities of the Eu-SnO_2_ device are 8.56, 3.92, 2.54, and 2.17, respectively, while the corresponding values of the pure counterpart are 1.36, 1.43, 1.81, and 1.54. Evidently, the responses of Eu-SnO_2_ sensor are much higher than those of the pure SnO_2_ one. It is worth stressing that, for acetone gas, the response has reached 8.56, much higher than the values of the other gases. It could be demonstrated that the dopant Eu can effectively improve the response of SnO_2_ NB.

Figure 5d displays the chemical resistance response of Eu-SnO_2_ NB and SnO_2_ NB sensors to different gas concentrations at 210 °C. With the concentration climbing up, the response/recovery time of Eu-SnO_2_ NB (SnO_2_ NB) sensor takes the values of 8/9 (5/7), 10/11 (12/14), 11/14 (12/13), 14/16 (14/16), and 15/19 (15/16) s. Their values are actually more or less the same in size. The detection lasted a few months and was repeated over and over again. Although during the period, the humidity ranged from 30 to 70 RH%, there is almost no fluctuation in the response, which could demonstrate that humidity has no effect on the sensor’s performance.

**Fig. 6:**
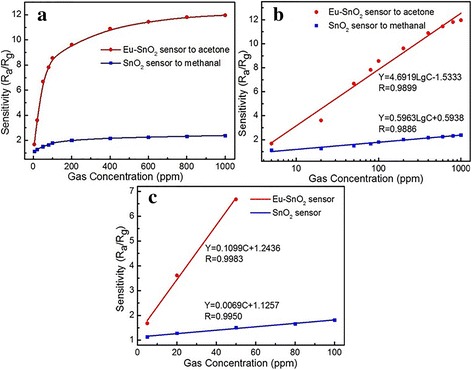
The curves of **a** response versus gas concentration, **b** response versus the logarithm of the concentration, and **c** response versus gas concentration in low range for the two sensors

We plotted the curves of the response of the two sensors and gas concentration at 210 °C, as shown in Fig. 6a. The gradient decreases with the increase in gas concentration may be caused by the increasing surface coverage by the adsorbed molecules [23]. As shown in Fig. 6b, the response versus the logarithm of the concentration can be well fitted by a straight line. From that, the sensitivity coefficients of Eu-SnO_2_ and SnO_2_ sensors could be calculated and the results are 4.6919 and 0.5963, indicating that Eu doping could improve the gas sensing performance effectively.

**Fig. 7:**
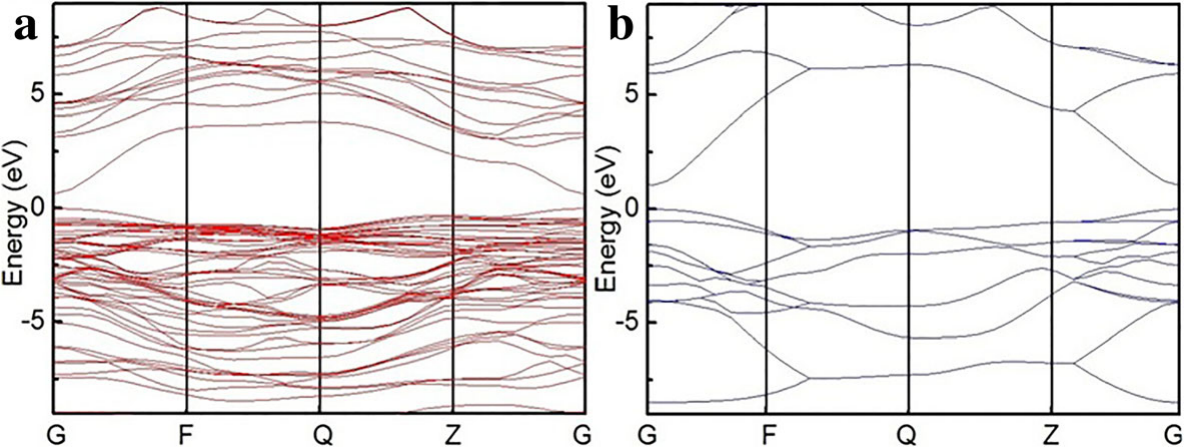
Band structure of **a** Eu-SnO_2_ and **b** SnO_2_

The fitting curves of the sensitivity versus gas concentration in low scales are presented in Fig. 6c. It reveals that the slopes are 0.1099 and 0.0069, respectively. The theoretical detection limit (TDL) of the sensor can be derived from the root-mean-square deviation $\left(\mathrm{RMSD}=\sqrt{{\mathrm{S}}^2/\mathrm{N}}\right)$, where N is the number of the selected points at the baseline in Fig. 5d and S is the standard deviation of these points [24]. The TDLs of Eu-SnO_2_ NB and SnO_2_ NB sensors can be calculated based on TDL (ppm) =3× (RMSD/slope) with the signal-to-noise ratio of 3 [25], and the results are 131 and 230 ppb. To understand the mechanism of the above observation, the calculation of the band structure of SnO_2_ and Eu-SnO_2_ was needed. As shown in Fig. 7, the top of the valence band and the bottom of the conduction band are located at point G in the Brillouin zone and it means that SnO_2_ is a direct-band gap semiconductor with a band gap of 1.047 eV. The calculated band gap is lower than the experimental value of 3.6 eV, which is due to the use of DFT. After Eu doping, the bottom of the conduction band moves to lower energy, so the band gap is narrowed down to a value of 0.636 eV. As a result, the needed energy for the electrons jumping from the valence band to the conduction band becomes smaller, the electron excitation being easier, a red-shift occurring in the absorption band, the range of spectral response expanding, and the efficiency of electron excitation could be improved. In one word, Eu doping improves the electrochemical properties of SnO_2_.

**Fig. 8:**
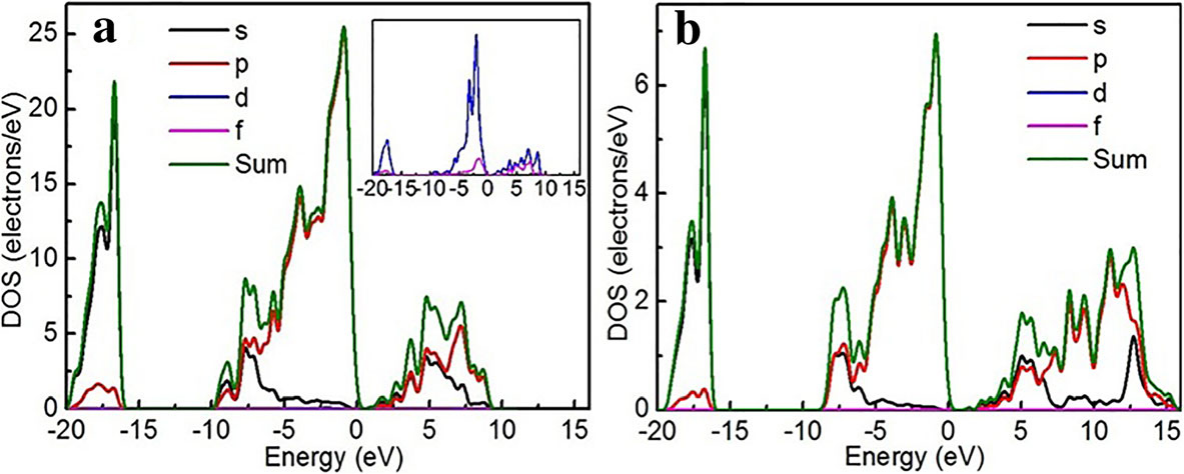
Density of states of **a** Eu-SnO_2_ and **b** SnO_2_

Figure 8 shows the density of states of Eu-SnO_2_ and SnO_2_, from which some changes caused by Eu doping can be observed. It shows that the low-energy parts (−20~0 eV), which are mainly composed of Sn 5s and O 2p states, are less influenced by Eu doping. As the inset of Fig. 8a shows, d and f orbits produce three peaks after Eu doping, and this means that there has appeared the impurity levels. As a result, the band gap becomes narrower, which could lead to an improvement in the conductive performance of SnO_2_.

**Fig. 9:**
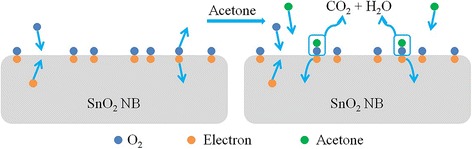
The schematic diagram of gas-sensing mechanism

As a metal oxide material, SnO_2_-based sensor belongs to the surface-controlled type [26]. The schematic diagram of the gas-sensing mechanism is shown in Fig. 9. Upon being exposed to air, the oxygen will be adsorbed on the surface, trapping free electrons, which could result in the formation of the depletion layer and the decline in conductivity according to Eq. 1

1 $$ {\mathrm{O}}_2 + {\mathrm{e}}^{-}\to {\mathrm{O}}^{\mathrm{x}} $$

where O^x^ means all kinds of oxygen ions [27, 28].

It is suggested that the oxygen-negative ions will react with the injected target gases and release the captured electrons back to the electron-depleted regions, reducing the resistance following these reactions [29, 30]

2 $$ \mathrm{C}{\mathrm{H}}_3\mathrm{C}\mathrm{O}\mathrm{C}{\mathrm{H}}_3+{\mathrm{O}}^{\mathrm{x}}\to \mathrm{C}{\mathrm{O}}_2 + {\mathrm{H}}_2\mathrm{O} + {\mathrm{e}}^{-} $$

3 $$ \mathrm{HCHO} + {\mathrm{O}}^{\mathrm{x}}\to \mathrm{C}{\mathrm{O}}_2 + {\mathrm{H}}_2\mathrm{O} + {\mathrm{e}}^{-} $$

Eventually, due to the trapping and release of electrons, the conductivity of nanobelt generates an evident change and achieves the sensing improvement. Besides, the performance of the doped sensor is much higher than that of its counterpart. Therefore, it is possible that Eu plays a significant role. According to the theoretical results, Eu doping could improve the electrochemical properties and conductive performance of SnO_2_. Then, the improved properties could contribute to a more rapid increase in the number of free electrons, narrow the electronic depletion layer, and enhance the deoxidation reaction on the surface. Just as a catalyst, Eu ions could promote the reactions around them [31]. Moreover, the possible reactions caused by Eu have been presented as below [32]:

4 $$ \mathrm{E}{\mathrm{u}}^{3+} + {\mathrm{H}}_2\mathrm{O}\ \to\ \mathrm{E}\mathrm{u}{\mathrm{O}}^{+} + {\mathrm{H}}^{+} $$

5 $$ \mathrm{E}\mathrm{u}{\mathrm{O}}^{+} + {\mathrm{O}}^{\mathrm{x}}\to\ \mathrm{E}{\mathrm{u}}_2{\mathrm{O}}_3 + {{\mathrm{V}}_{\mathrm{O}}}^{\bullet \bullet } + {\mathrm{e}}^{-} $$

According to Eqs. 4 and 5, more defects will be formed when Eu ions replace the position of Sn atoms in SnO_2_ lattice, and this could lead to more active reactions at the same time. In addition, Eu doping can trigger the dehydrogenation which can lower down the energy of the redox reactions [33]. Through these ways, Eu realizes the boost of sensor performance.

## Conclusions

The Eu-doped and pure SnO_2_ NBs with regular morphology and great flakiness ratio have been fabricated and the relevant single nanobelt devices have been prepared. Certainly, their electrical- and gas-sensing properties have been investigated and it is found that the conductivity of Eu-SnO_2_ is higher than that of the pure one. The results of their sensitive measurements show that the optimum working temperatures of them are both 210 °C, and the highest sensitivity of Eu-SnO_2_ device to 100 ppm of acetone is 8.56, which is 6.29 times as large as that of its pure counterpart (1.36). The response recovery time of the two devices is less than 20 s. The TDL of the Eu-SnO_2_ NB and SnO_2_ NB sensors have been calculated, and the results are 131 and 230 ppb, respectively. The theoretical results have proved that Eu doping could improve the electrochemical properties and conductive performance of SnO_2_. All the results reveal that Eu doping could improve the sensitivity of sensing response of SnO_2_ NB, especially to acetone gas.
